# Effectiveness of the innovative 1,7-malaria reactive community-based testing and response (1, 7-mRCTR) approach on malaria burden reduction in Southeastern Tanzania

**DOI:** 10.1186/s12936-020-03363-w

**Published:** 2020-08-14

**Authors:** Yeromin P. Mlacha, Duoquan Wang, Prosper P. Chaki, Tegemeo Gavana, Zhengbin Zhou, Mihayo G. Michael, Rashid Khatib, Godlove Chila, Hajirani M. Msuya, Exavery Chaki, Christina Makungu, Kangming Lin, Ernest Tambo, Susan F. Rumisha, Sigsbert Mkude, Muhidin K. Mahende, Frank Chacky, Penelope Vounatsou, Marcel Tanner, Honorati Masanja, Maru Aregawi, Ellen Hertzmark, Ning Xiao, Salim Abdulla, Xiao-Nong Zhou

**Affiliations:** 1 National Institute of Parasitic Diseases, Chinese Center for Disease Control and Prevention, 207 Rui Jin Er Road, Shanghai, 200025 People’s Republic of China; 2 Ifakara Health Institute, P. O. Box 78378, Kiko Avenue, Mikocheni, Dar es Salaam, Tanzania; 3 Swiss Tropical and Public Health Institute, Basel, Switzerland; 4 University of Basel, Basel, Switzerland; 5 Guangxi Center for Disease Control and Prevention, Nanning, China; 6 Higher Institute of Health Sciences, Université des Montagnes, Bangangté, BP 208 Cameroon; 7 National Malaria Control, Ministry of Health, Community Development, Gender, Elderly and Children, Dodoma, Tanzania; 8 National Institute for Medical Research (NIMR), P.O. Box 9653, Dar es Salaam, Tanzania; 9 The Global Malaria Programme (GMP), World Health Organization, Geneva, Switzerland; 10 Department of Global Health and Population, Harvard T.H. Chan School of Public Health, Boston, MA USA

**Keywords:** Malaria, 1,7-mRCTR approach, Community-based, Testing, Treatment, Response, Health facilities, Control, Intervention, Surveillance

## Abstract

**Background:**

In 2015, a China-UK-Tanzania tripartite pilot project was implemented in southeastern Tanzania to explore a new model for reducing malaria burden and possibly scaling-out the approach into other malaria-endemic countries. The 1,7-malaria Reactive Community-based Testing and Response (1,7-mRCTR) which is a locally-tailored approach for reporting febrile malaria cases in endemic villages was developed to stop transmission and *Plasmodium* life-cycle. The (1,7-mRCTR) utilizes existing health facility data and locally trained community health workers to conduct community-level testing and treatment.

**Methods:**

The pilot project was implemented from September 2015 to June 2018 in Rufiji District, southern Tanzania. The study took place in four wards, two with low incidence and two with a higher incidence. One ward of each type was selected for each of the control and intervention arms. The control wards implemented the existing Ministry of Health programmes. The 1,7-mRCTR activities implemented in the intervention arm included community testing and treatment of malaria infection. Malaria case-to-suspect ratios at health facilities (HF) were aggregated by villages, weekly to identify the village with the highest ratio. Community-based mobile test stations (cMTS) were used for conducting mass testing and treatment. Baseline (pre) and endline (post) household surveys were done in the control and intervention wards to assess the change in malaria prevalence measured by the interaction term of ‘time’ (post vs pre) and arm in a logistic model. A secondary analysis also studied the malaria incidence reported at the HFs during the intervention.

**Results:**

Overall the 85 rounds of 1,7-mRCTR conducted in the intervention wards significantly reduced the odds of malaria infection by 66% (adjusted OR 0.34, 95% CI 0.26,0.44, p < 0001) beyond the effect of the standard programmes. Malaria prevalence in the intervention wards declined by 81% (from 26% (95% CI 23.7, 7.8), at baseline to 4.9% (95% CI 4.0, 5.9) at endline). In villages receiving the 1,7-mRCTR, the short-term case ratio decreased by over 15.7% (95% CI − 33, 6) compared to baseline.

**Conclusion:**

The 1,7-mRCTR approach significantly reduced the malaria burden in the areas of high transmission in rural southern Tanzania. This locally tailored approach could accelerate malaria control and elimination efforts. The results provide the impetus for further evaluation of the effectiveness and scaling up of this approach in other high malaria burden countries in Africa, including Tanzania.

## Background

In recent decades, there has been a substantial increase in financial and political commitment supporting the fight against malaria. Specifically, investments have gone into the scaling-up of vector control tools such as long-lasting insecticidal nets (LLINs) and indoor residual spraying (IRS) [1–6]. Additionally, significant improvements have been made in case management by the introduction of artemisinin-based combination therapy (ACT) [7, 8]. Such interventions have produced a massive reduction in the malaria burden and prevented several million deaths worldwide [1, 9, 10]. Globally, it is estimated that 228 million malaria cases were reported in 2018, with Africa bearing the brunt of this burden [1]. Over the last two decades, malaria control programs in Tanzania have become larger and more widespread, with a national scale-up of preventive strategies and improved quality and access to testing and treatment [8, 11, 12]. As a result, the prevalence has declined from an average of 18.1% in 2008 to 7.3% in 2017 [13, 14]. Despite these notable achievements, the fight is far from over. More than 93% of the Tanzanian population remains at risk of malaria [11, 14]. Sustaining the gains and making progress towards elimination remain the main challenges owing to financial gaps to ensure universal coverage, access to health services, and epidemiological challenges.

To guide malaria elimination, the World Health Organization (WHO) has released the *Global Technical Strategy for Malaria 2016*–*2030*, which emphasizes the importance of transforming the malaria surveillance response strategy into a core intervention [15]. The national malaria control programme (NMCP) is advocated to take into account the epidemiology and diversity of malaria in each country using malaria burden stratification, and tailoring interventions to the local context [15, 16]. Likewise, WHO’s Test-Treat-Track (T3) [17] initiative for malaria surveillance and the response has been in place to guide the goals of universal coverage of preventive tools and eliminate malaria deaths and eradicate the disease. In China, professionals have developed the ′1-3-7′ approach [18, 19]. In this surveillance system, confirmed cases must be reported within one (1) day, origin (imported or domestic) investigated within three (3) days, and appropriate intervention to reduce the chance of onward transmission must be done within seven (7) days. This highly personnel-intensive approach has been used in China’s national malaria elimination programme [20], with the effect of near-elimination of domestic cases [1, 21]. Several studies have shown that targeted interventions could hasten malaria elimination [22–26]. However, the question remains open regarding what intervention optimization strategies are applicable and what would be the best model to introduce intervention in higher- transmission settings.

In Tanzania, a review of the most recent population-based malaria indicator survey and health facility (HFs) information has shown the high heterogeneity of malaria endemicity within regions across the country [11, 27–30], underscoring the need to deploy appropriate interventions carefully. New approaches for malaria control are needed to sustain and accelerate progress towards elimination, and synthesis of the WHO-T3 initiative and the Chinese experience of surveillance and response offers a great opportunity for the identification of new approaches.

The main objectives of the China-UK-Tanzania tripartite pilot project were: (i) to reduce the malaria burden by 30% in 2018 compared to that of 2015; (ii) to strengthen the capacity of malaria control at the local level; and, (iii) to explore the appropriate model and mechanism to develop scalable anti-malarial programmes for Tanzania and other African countries. Taking the cues from China’s domestic success and the WHO-T3 initiative, the Chinese and Tanzanian teams jointly developed a new approach for malaria surveillance and response. The 1,7-malaria reactive community-based testing and response (1,7-mRCTR) approach operates at the village level. It entails reporting of any confirmed malaria cases at the HFs within 24 h combined with a follow-up the next week consisting of focal treatment of holo-endemic villages to stop transmission at the same phase of the *Plasmodium* life-cycle. This targeted approach aligns with the WHO’s high-impact initiative for countries with moderate and high transmission [31], tailoring the Chinese experiences and the WHO-T3 initiative into the local settings of Tanzania. The overriding objective of this particular paper is to establish the effectiveness of the 1,7-mRCTR approach by observing changes in community-level malaria prevalence, by comparing changes from baseline to endline surveys within and between study areas, in areas where the burden of malaria infection is high. As a secondary objective, the changes in malaria incidence reported at the health facilities after interventions in the villages were also studied.

## Methods

### Study design and setting

**Fig. 1:**
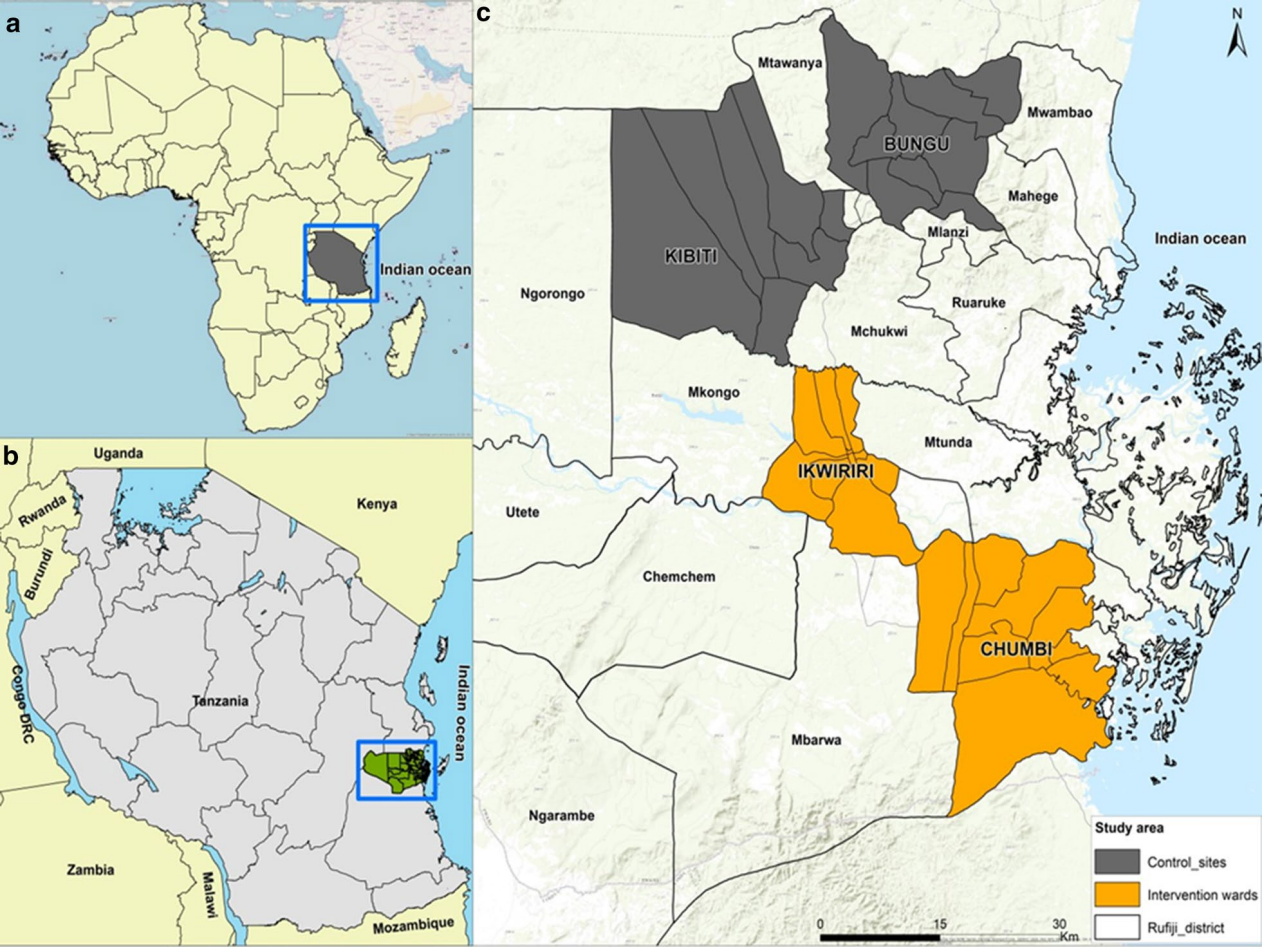
Location of the study area in, Rufiji district, Tanzania. **a** Overview map of Africa showing Tanzania location, **b** overview location of Rufiji District in Tanzania, **c** overview map of Rufiji district indicating the intervention (Ikwiriri and Chumbi) and control wards (Bungu and Kibiti). Base Map was obtained from OpenStreetMap through the ArcGIS plugin

The study area was the Rufiji District, located in southeastern Tanzania, which has been described previously [32, 33]. A pilot project was implemented from September 2015 to June 2018. Two control wards (Bungu and Kibiti) and two intervention wards (Chumbi and Ikwiriri) were selected. Based on the malaria incidence rates recorded the preceding year by staff at the local HFs, each arm contained one high-transmission and one low transmission-ward. In this study, malaria incidence < 20/1000 cases and ≥ 20/1000 cases were considered as low and high transmission wards, respectively. Since these wards (except Chumbi) had been part of the previous Health and Demographic Surveillance System site (HDSS), they were considered well prepared for testing and treatment evaluation of the proposed model under programme conditions [34]. The two control wards received no interventions beyond what was provided by the NMCP, primarily LLINs. Fourteen facilities were located in the control wards, eight in Bungu and six in Kibiti, but only one per ward was a proper health centre, the others being dispensaries. The intervention wards contained 11 HFs covering 18 villages, again with one proper health centre per ward, and the rest being dispensaries. Nearly 89% of the people in Rufiji live within 5 km of an HF [35]. Since the approximate distance between the centres of Ikwiriri (intervention ward) and Kibiti (control ward) was 30 km, it is unlikely that people from the control wards attended the screenings in the intervention wards. Based on the census of 2012, the total populations of the intervention and control wards in 2012 were 72,163 and 53,292, respectively [36]. The average household size in Rufiji was 4.4, and 45% of the total population was under 15 years of age [36]. Figure 1 shows a map of the study area with the location of the pilot project wards.

### Intervention

Implementation of the project started with workshops and kick-off meetings held by expert teams from both implementing partner countries in China to share and exchange knowledge on malaria control and experiences from the two countries, which also involved field visits to both Tanzania and China. The visit involved consultations with central and local government authorities for an understanding of health system issues related to the provision of malaria services and identification of project sites. Subsequently, technical review of the Tanzanian health information systems and health facility-based malaria service provision both revealed huge variations in terms of the data structure (individual versus aggregated case reporting), timeliness and completeness of the information, the precision of the information in terms of mapping the precise location of the malaria cases to allow for finer microstratification down to the sub-district level for targetting the intervention and to top it all the variation in malaria burden. While 1-3-7 is best suited for very low transmission areas with a relatively very low number of cases, the pilot project was to be implemented in a moderate transmission site with a huge burden of malaria, hence the decision to adopt the 1,7-mRCTR approach.

The locally tailored 1,7-mRCTR surveillance and response approach was the main intervention in the intervention communities, in addition to the existing malaria control prevention implemented by the Ministry of Health through NMCP. While the package of this project was deployed the intervention arm only, the existed health intervention by the government through the ministry of health continued equally in both arms. LLINs are the main malaria vector control in the district. In May 2016, LLINs were distributed in the district through Universal Coverage Campaign (UCC). Besides community screening and treatment, the 1,7-mRCTR approach included quality control supervision of case detecting capability through increasing parasitological examination rate of all suspected malaria cases at the corresponding community-level.

All village members through community health education campaigns were advised to seek treatment at a health facility for any febrile illness. Furthermore, information, education, and communication (IEC) materials were developed purposely with local-tailored key messages for the targeted community.

Within both the intervention and control wards, data quality assurance and malaria service availability and provision surveys were regularly conducted by the NMCP and Council Health Management Team (CHMT) as an integral part of their mandate and responsibility. The project team had special emphasis on the intervention sites and communicated any deficiencies observed to either the CHMT or NMCP for correction. On a random day of the week, the quality control team conducted a spot check survey at HFs to cross-check the quality, accuracy, and consistency of data and status of malaria supplies (diagnostics and antimalarials). These spot check visits were envisaged to increase the ensure compliance of the service providers to the standard operating procedures for malaria service provision at HFs as well as validate the quality of the data being submitted and used for decision making.

Weekly, all malaria suspects presenting to local HFs were tested for malaria by RDT or microscopy, were allocated to the villages of patient(s) residence. The response was mounted in the village with the highest ratio of the number of confirmed malaria positive cases/the number of suspects. There was no specified cutoff. The highest village specific malaria incidence ratio varied with time. Monday-Friday of the following week teams of community-based health care workers (CHCWs) set up community-based mobile test stations (cMTS) in different hamlets (sub-villages) of the ‘hot spot’ village, starting with those presumed to have the highest case ratio, but moving around to ensure the village-wide coverage of community members. The detailed activities for the 1,7-mRCTR implementation are provided in the Additional file 1 and study protocol which has been published elsewhere [33].

### Implementation

Before the study began, formal meetings were held between the researchers, the District Medical Officer’s (DMO’s) office, the CHMT staff, and local community leaders to discuss the study objectives, procedures, and timelines. Accompanying printed materials in *Swahili* were distributed at this meeting to provide complementary project information. To maximize project acceptance after a village had been identified as a hotspot, weekly social mobilizations were initiated, i.e., the field supervisor and village community leaders held meetings to discuss the logistics and cMTS locations. Upon deciding on the locations, village leaders and CHCWs informed the rest of community members about the presence of the cMTS, emphasizing that testing and treatment were free. Although only the hotspot villages were targeted, people from neighbouring villages within the intervention sites, who came for testing were also tested and treated. When a village re-appeared as a hotspot, test station locations were chosen using information based on the previous time(s) of response.

**Fig. 2:**
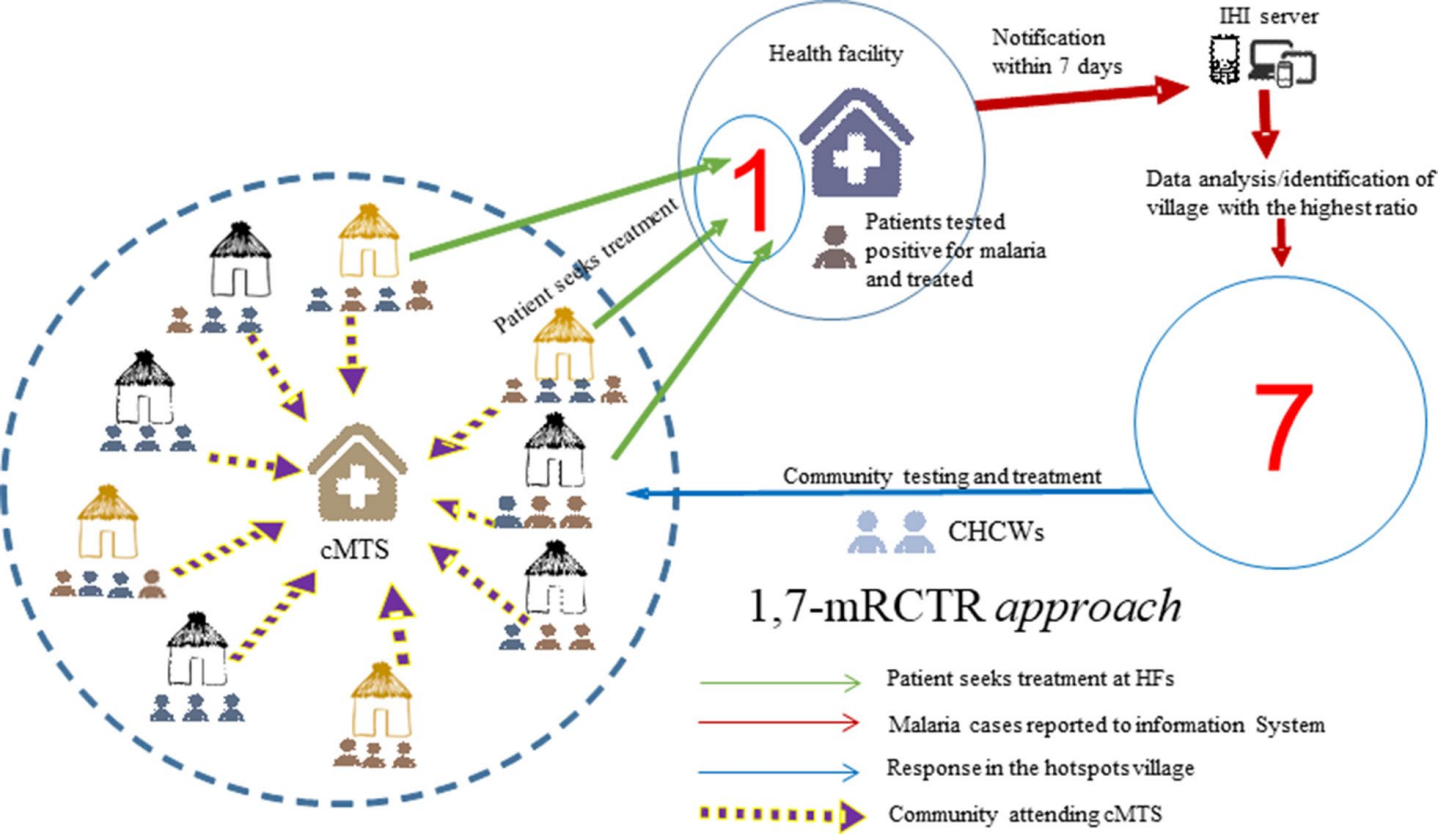
Diagrammatic representation of the 1,7-mRCTR approach as implemented in the intervention arm

Village members above 6 months of age were invited to be screened for malaria. On the day of screening, the participant’s informed consent was taken and registered. Finger-prick blood was collected from participants and used for both RDTs (CareStartTM Malaria Pf/PAN (HRP2/pLDH) Ag Combo RDT) and blood slides to test malaria parasitaemia. For prompt treatment, only RDTs results were used, and, if positive, treated with dihydroartemisinin-piperaquine (D-ARTEPP) following the national policy guidelines for malaria treatment [37]. The blood slides were used for quality control, and to determine the malaria species [32]. In case of complications or severe cases, the participants were referred to the nearby health facilities. The participants’ demographic information, travel history for the previous two weeks, medical histories such as medications taken, and vital signs were also recorded. Due to security problems in the study area, the activities stopped for 8 months from January to August 2017 and resumed from September 2017 to April 2018. Figure 2 illustrates the schematic diagram of the 1,7-mRCTR implementation in the intervention area.

### Evaluation

**Fig. 3:**
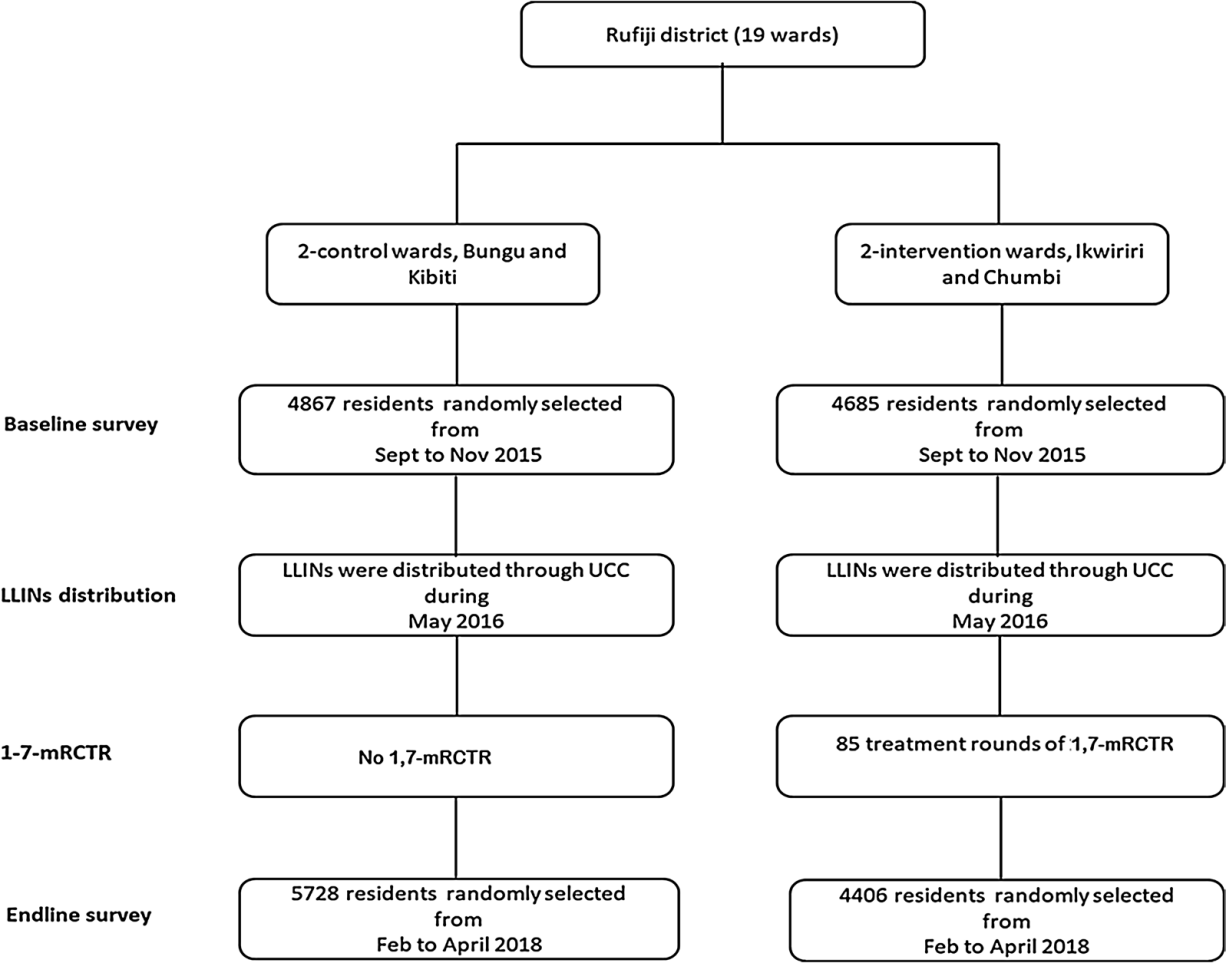
Schematic diagram of the study design, intervention activities, and the number of participants sampled for baseline and endline cross-sectional household surveys for the 1,7-mRCTR approach evaluation

The primary measure of the effectiveness of 1,7-mRCTR, determined in advance, was the adjusted comparison of the changes in malaria prevalence from before the project to after the intervention in the control and intervention areas. This was a non-experimental study, the entire evaluation was based on the baseline and endline household cross–sectional surveys with independent samples conducted in both intervention and control areas. Figure 3 shows the number of participants sampled for baseline and endline cross-sectional household surveys for the 1,7-mRCTR approach evaluation.

The baseline was created using data collected from September to November 2015, with the endline survey done from February to April 2018. A random sample of 2000 households was selected based on community census data for each of the baseline and endline surveys. The sample size and power calculation for this evaluation can be found in the previously published protocol [33]. A structured questionnaire was designed based on the standard RBM-MERG guidelines with modification to fit the study area [38]. It was developed in English, translated into *Swahili,* and installed on tablets using the Open Data Kit software. A full description of the study’s aim and the objective was given to the head of the household at the first visit. All participants were provided with a written informed consent form describing the risks, benefits, and the participant’s rights to free diagnosis and treatment. The right to refuse participation without penalty was explained and guaranteed. If a household in the list could not be located or did not wish to participate, a nearby house with similar features was selected for replacement. At the household level, each occupant present was tested in situ for parasite infection using an RDT, blood smear, and filter papers. Only RDT results were considered in the analysis. Other people were not pricked because they only accepted to participate in the interviews without consenting to invasive procedures necessary for blood collection. However, this was not considered a serious problem that could bias the study because it was expected and addressed during the design stage where 20% of the calculated sample was added. The detailed methods for the baseline survey have been reported previously [32].

### Statistical analysis

Baseline and endline prevalences were computed as the values of the intercepts in generalized estimating equations (GEE) clustering on household and using the identity link, with their standard errors. Univariate analyses were done to test the relationships between different potential explanatory variables and prevalent malaria. Comparisons between the intervention and control arms were done similarly for each survey. Malaria prevalence was modelled using GEEs with the logit link, clustering at the household level. The effect of 1,7-mRCTR was estimated by comparing the changes from the baseline malaria prevalence to that at the endline surveys (main effect ‘time’) in the two areas (main effect ‘area’), using the interaction term of area and ‘time’ (baseline vs endline) as the measure of programme effect (difference-of-differences). When the interaction effect is included in the model, the main effect of the area describes the difference between the two areas at baseline, and the main effect of time gives the ‘average’ change in odds of malaria from baseline to endline. The interaction effect represents the difference between the changes in the two areas.

Categorical variables were presented as numbers (percentages), while continuous variables were presented as mean (confidence interval)/medial (quartile range), respectively. Potential confounders included age (categorized as under 5 years, 5 to 14 years and above 15 years), sex, LLINs use the previous night, and socio-economic status (SES). The wealth index (SES) as a potential risk factor for malaria infection was generated using principal component analysis on a list of assets possessions to produce the SES quintiles [39].

For the duration of the project, routine data were available only for the intervention wards, and the only routinely collected case-related numbers were in HFs. Therefore, the case ratios (HFs cases/population) were analysed rather than true incidence values. A mixed-effects regression model with the village as a random effect was used to analyse the impact of the 1,7-mRCTR in reducing HFs case ratios between villages receiving malaria intervention and those not receiving it. The detailed analytical procedure for the health facility data analysis is described in Additional file 2. Statistical analyses were performed using STATA software (version 15.1, College Station, TX, USA) and SAS software (version 9.4, Cary, NC, USA).

## Results

### Community impact of 1,7-mRCTR on the reduction of malaria prevalence

Overall 9522 and 10,134 participants were surveyed during the baseline and endline surveys, respectively. A total of 7691 and 7989 individuals agreed to provide finger-prick blood for malaria testing for each respective survey (Table 1). The median household size for the baseline and endline survey population was 6 (interquartile range IQR 4–8) and 5 (IQR 4–7), respectively. All age groups were included in the study, and people ≥ 15 years of age accounted for more than 52% of all participants, followed by the 5–15 years age group (31%). In both surveys, females accounted for 55% of the total surveyed participants. The disease burden recorded in both intervention and control wards at the baseline survey significantly declined by the time of the endline survey. Malaria prevalence in the intervention wards declined by 81% (from 26.0% (95% CI 23.7–27.8), at the baseline to 4.9% (95% CI 4.0–5.9) at the end of the study) (Table 1). In the control wards, malaria prevalence was reduced by 52% from 28.1% (95% CI 26.1–30.2) at the baseline to 13.4% (95% CI 12, 12–14.7) at the endline survey. Both intervention and control wards showed a significant increase in LLIN use over the time of the study as a whole. In the intervention wards, the use of LLINs increased from 66% (95% CI 62.6–69.1) at the baseline to 83% (95% CI 81.3–85.3) at the final survey. In the control wards, the use of LLINs increased from 49.4% (95% CI 46.4–52.4) at the baseline survey to 80% (95% CI 77.9–81.5) at the end.

**Table 1:** Demographic and characteristics of participants in the baseline and endline community surveys

Characteristics	Baseline survey		Endline survey	
	Control	Intervention	Control	Intervention
Population^a^, *n (%)*	4867	4685	5728	4406
Age group, years				
< 5	908 (18.7%)	852 (18.2%)	986 (17.2%)	702 (15.9%)
5–15	1602 (32.9%)	1425 (30.4%)	1727 (30.2%)	1307 (29.7%)
> 15	2357 (48.4%)	2408 (51.4%)	3015 (52.6%)	2397 (54.4%)
Gender, n (%)				
Female	2698 (55.4%)	2509 (53.6%)	3310 (57.8%)	2464 (55.9%)
Male	2169 (44.6%)	2176 (46.4%)	2418 (42.2%)	1942 (44.1%)
Malaria infection^a^, n (%, 95% CI)				
Positive	1103 (28.1, 26.1–30.2)	967 (25.7, 23.7–27.8)	621 (13.4, 12.12–14.7)	163 (4.9, 4.0–5.9)
Negative	2827 (71.9, 69.9–73.9)	2794 (74.3, 72.2–76.3)	4029 (86.6, 85.3–87.9)	3176 (95.1, 94.1–96.0)
Bed-net use^c^, n (%, 95% CI)				
Yes	2316 (49.4, 46.4–52.4)	2969 (65.9,62.6–69.1)	4568 (79.7, 77.9–81.5)	3673 (83.4, 81.3–85.3)
No	2375 (50.6, 47.6–53.6)	1534 (34.1,30.9–37.4)	1160 (20.3, 18.5–22.2)	733 (16.6, 14.7–18.7)

^a^Number of individuals surveyed

^b^based on malaria rapid testing using RDT

^c^Reported insecticide-treated bed-net use the previous night

### Multivariate analysis

Multivariate analysis using GEEs is presented in Table 2. The baseline malaria prevalence was lower in the intervention wards, adjusted odds ratio (aOR) 0.41 (95% CI 0.35–0.48, p < 0.001), and both wards had much lower odds of malaria at endline compared to baseline, aOR 0.90 (95% CI 0.77–1.04, p = 0.14) (Table 2). The aOR of the endline/baseline was 0.34 (95% CI 0.26–0.44, p < 0.001). The decline in prevalence odds in the intervention wards was much greater than that in the control wards. LLIN use was associated with significantly lower odds of having malaria: aOR 0.71(95% CI 0.63–0.80). The highest wealth quintiles (i.e., those better off) people were less likely to be infected by malaria, aOR 0.21 (95% CI 0.17–0.26, p < 0.001)) as compared to the lowest (i.e., the poorest). The 5–15 years old participants had twice as high odds of malaria infection compared to those under five, aOR 2.13 (95% CI 1.89–2.40, p < 0.001)) (Table 2).

**Table 2:** Univariate and multivariable analysis describing the effects of the 1,7-mRCTR and risk factors for malaria infection

Variables	Univariable model		Multivariable model	
	COR (95% CI)	p-value	aOR (95% CI)	p-value
Survey years				
Baseline	1 (ref)	–	1 (ref)	–
Endline	0.29 (0.26–0.33)	< 0.001	0.41 (0.35–0.48)	< 0.001
Site				
Control wards	1 (ref)	–	1 (ref)	–
Intervention wards	0.74 (0.66–0.84)	< 0.001	0.90 (0.77–1.04)	0.14
Comparison of endline to baseline				
Control	1 (ref)	–	1 (ref)	–
Intervention	0.17 (0.14–0.21)	< 0.001	0.34 (0.26–0.44)	< 0.001
Gender				
Female	1 (ref)	–	1 (ref)	–
Male	1.44 (1.32–1.57)	< 0.001	1.24 (1.13–1.36)	< 0.001
Age group, years				
<5 years	1 (ref)	–	1 (ref)	–
5–15 years	2.09 (1.87–2.34)	< 0.001	2.13 (1.89–2.40)	< 0.001
>15 years	0.67 (0.60–0.76)	< 0.001	0.67 (0.59–0.76)	< 0.001
Bed-net use^a^				
No	1 (ref)	–	1 (ref)	–
Yes	0.43 (0.38–0.48)	< 0.001	0.71 (0.63–0.80)	< 0.001
Wealth index				
Lowest	1 (ref)	–	1 (ref)	–
Second	0.86 (0.74–1.02)	0.076	0.75 (0.64–0.88)	< 0.001
Middle	0.62 (0.52–0.73)	< 0.001	0.56 (0.47–0.66)	< 0.001
Fourth	0.55 (0.46–0.65)	< 0.001	0.50 (0.42–0.60)	< 0.001
Highest	0.23 (0.19–0.29)	< 0.001	0.21 (0.17–0.26)	< 0.001

*cOR* crude odds ratio

*aOR* adjusted odds ratio

*CI* confidence interval

^a^Insecticide-treated bed-net use previous night

### Changes noted in the intervention communities

The Chumbi high-transmission ward had a total population of 26,631 people (per census), with 15,317 malaria cases (reporting to the HFs) during the study period. The Ikwiriri moderate-transmission ward had a total population of 45,532 people (per census), with 21,254 reported HF malaria cases (Table 3). The average case ratios (total number of positive cases per total population) were 5.34 and 4.38 (per 1000 population per week) for Chumbi and Ikwiriri, respectively. While both wards had roughly the same case ratio in the low transmission season (August-April), they diverged in the high transmission season (May–July), with a more considerable increase in Chumbi. A total of 50 rounds of 1,7-mRCTR visits were conducted in Chumbi, during which 6511 cases were treated. In the Ikwiriri ward, 35 rounds of 1,7-mRCTR visits were conducted, with 2924 cases treated. The median age of the participants subjected to the 1,7-mRCTR rounds was 15 years (IQR 7–28). One village never received a 1,7-mRCTR. No important adverse reactions were reported during the study period.

**Table 3:** Characteristics of participants screened and number of health facility cases and case ratios by ward, season and year during the 1,7-mRCTR project in the intervention wards

Characteristics	Moderate-transmission ward (Ikwiriri)	High-transmission ward (Chumbi)	Overall
Total population, n	45,532	26,631	72,163
Number of treatment rounds	35	50	85
Population screened	17,160	21,246	38,406
Malaria infection (%)^a^	2924 (17.0)	6511 (30.6)	24.57
Fraction of village population tested (mean (standard error))	10.5 (1.3)	12.0 (1.7)	11.4 (1.1)
Fraction of those tested who were positive	17.5 (1.7)	31.8 (2.6)	25.9 (1.8)
Total number of health facility cases, n	21,254	15,317	36,571
Number of health facility cases, (n (Weekly case ratio/1000 popn) (%))			
Low transmission season^b^ 2016	7728 (4.47)	5180 (3.96)	12,908 (4.25)
2017	5578 (3.22)	2825 (2.16)	8403 (2.77)
High transmission season^c^ 2016	4127 (6.47)	4049 (8.40)	8176 (7.30)
2017	3821 (5.99)	3263 (6.77)	7084 (6.33)

*popn* population

*std err* standard error

^a^Tested positive for malaria infection by RDT

^b^September to April

^c^May to August

There was a substantial decrease in weekly case ratios per 1000 population from 2016 to 2017 during both the low and the high season (Table 3). Weekly case ratios from 2016 to 2017 decreased proportionately more in the low season (Table 3). In Ikwiriri, the case ratio during the high season barely decreased at all (6.5 to 6.0%,), while in Chumbi the case ratio decreased proportionately less in the high season (8.4 to 6.8%, a 19.4% decrease) than in the low season (4.0 to 2.2%, a 45% decrease).

### Changes in reported HFs malaria cases at the village level

A mixed-effect regression model analysis of the routine HFs data controlling for the season, wards, their interaction and number of times the village was previously treated indicated that in the week after a 1,7-mRCTR visit in the village, the case ratio decreased by over 15.7% (95% CI − 33, 6) but was not significant (Table 4). From 2 to 5 + weeks after village treatment, the case ratio varied among weeks but was mostly below that during the week of treatment. The analysis separating the two intervention wards (Chumbi and Ikwiriri) showed the same trend of low-level case ratio reductions.

**Table 4:** Estimated change in malaria incidence case ratios compared to the hotspot week, by week after 1-7RCTR response in the village of the intervention wards

Weeks since treatment	Exchangeable model^a^			
	Estimate %	95% CI		p-value
	Ref			
Week of treatment	13.6	− 7.1	38.9	0.22
Week following treatment	− 15.7	− 33.0	5.9	0.14
2 weeks after treatment	− 3.1	− 24.5	24.3	0.80
3 weeks after treatment	5.3	− 17.9	35	0.69
4 weeks after treatment	9	− 18.5	45.8	0.56
5–13 weeks after treatment	8.7	− 7.1	27.2	0.30

^a^Based on a mixed model, weighted by the inverse probability of being in the designated week of or after the 1,7-mRCTR and controlling for ward, season, time since the beginning of the project

## Discussion

Surveillance is recognized as an intervention and considered instrumental in accelerating global malaria elimination efforts. However, all existing evidence to date does support the incorporation of surveillance as in intervention in low endemicity areas and no evidence comes from moderate to high endemicity areas. 1-3-7 model which inspired the development of this project and subsequent adopting of the 1,7-mRCTR approach, is a unique approach to implementing the recommended WHO-T3 and surveillance as an intervention for eliminating malaria. Nevertheless, the 1-3-7 model mainly worked in China whose target is elimination as opposed to Tanzania majority of which still experiences moderate to the high transmission and hence the target is to reduce the burden. The adopted 1,7 mRCTR has demonstrated beyond doubt that, a locally tailored surveillance-response strategy can successfully result in a dramatic reduction of disease burden and hence accelerate elimination efforts. The study offers the first attempt at establishing an appropriate surveillance-response model that will fit most African settings in driving the malaria elimination agenda.

The 1,7-mRCTR intervention substantially reduced the community malaria burden in the areas characterized by moderate to high malaria transmission in southeastern Tanzania. The dramatic reduction in the intervention wards (81%) compared to the control areas (52%) produced clear and practical evidence underlining the usefulness of the 1,7-mRCTR intervention, which was bolstered by the multivariate analysis showing that the reduction of the malaria prevalence (66%) was beyond the impact of LLINs alone. Importantly, current malaria interventions, including the most advanced ones using the novel vaccination approach, have only reported a 30–50% effect beyond that of LLIN use [40].

The results are consistent with other studies demonstrating the effect of early diagnosis and community treatment in reducing the burden of malaria infection in sub-Saharan African countries and elsewhere [26, 41–43]. However, contrary to these studies, the 1,7-mRCTR for screening and treatment was based on using health facility-based data to geographically map the patients and identify village as the index of observation, evaluation, and targeting instead of individuals. The advantage of this approach was that it provided a chance for all community members to participate, which is in line with the current WHO-recommended focus and strategy on the high burden and high impact [31]. Also, the fact that the 1,7-mRCTR involved local CHCWs provides a strong foundation for the sustainability of addressing the essential systemic key issue to project implementation. Furthermore, though it is slightly beyond the scope of this paper, the intervention has demonstrated capable of not only conferring protection to the beneficiary communities but also has delivered short term impact on the health system’s service provision by reducing the number of the hospital attendance in subsequent weeks following the intervention week. However this effect needs to be evaluated further to establish the exact magnitude and duration of effect.

Moreover, looking at the intervention design the success of the 1,7-mRCTR was mainly contributed by the daily collected and reviewed HFs data and used to identify weekly priority areas for priority screening and treatment by local CHCWs teams. This though tedious exercise, led to a positive outcome through effective engagement of all involved parties service providers to provide data and the communities to receive the intervention. This reduction of malaria burden using the 1,7-mRCTR approach does highlight the feasibility and opportunity of simply emphasizing using HFs data for microstratification of cases and devising appropriate accessible, prompt, and effective malaria interventions, especially in remote, underserved areas with moderate-high malaria transmission [40, 44, 45]. Furthermore, the involvement of the local CHCWs has been instrumental in optimizing the awareness, acceptability, and coverage of the intervention since most of the CHCWs were recruited and worked from the project area where they were familiar to geographical settings and the culture.

Despite the success demonstrated by the adopted approach in Tanzania, key lessons from the project team would be to be observant. While the combination of the WHO-T3 initiative and the Chinese 1-3-7 model could be easily taken up consideration must be made for locally tailoring in most malaria endemic settings across Africa requiring more modification. The disease epidemiology and the differences in health systems’ such as lack of proper individual tracking systems versus transmission status as well as the inability of existing information systems to allow for sub-district level microstratification would hamper the adaptability of such strategies in most settings. Therefore, as exemplified by our team the process of crystallizing and making some minimal essential adaptions, such as the inclusion of parameters allowing isolation (in the village of residence) of cases testing positive and the development of an electronic platform for individual cases reports is essential to ensure the success of the strategy. The modified platform was able to capture all individual malaria daily, map the individual patient down to the village level allowing for microstratification and assessment of the magnitude of the burden comparing the villages within the catchment area before launching the response by the team of CHCWs. It should be noted here that the 1,7-mRCTR approach was successfully deployed local community-based personnel, including field interviewers, nurses, laboratory technicians, under the oversight of clinicians who serviced the entire catchment area. This approach was adopted to address both the acceptability of the intervention as well as the shortage of human resources for health.

The findings from this study are limited in terms of spatial and temporal coverage. The project was implemented in only one district of the country, which has several other settings with varying epidemiological, ecological, socio-economic, and cultural. There is still a need to further explore whether this intervention package would lead to similar results in other areas, which are epidemiologically, ecologically, and socio-economically different. Extending this intervention in other settings could validate the findings of the pilot project and further build confidence in possible uptake by national programmes and subsequently scale-up for impact. Other potential limitations include that the study was a before-after assessment of which no adequate control of study participants is conducted which may compromise the strength of the evidence, also it was implemented in the area where other programmatic activities were going on as usual, which, despite our rigorous analysis some of the observed impacts might have been altered. Indeed, the 1,7-mRCTR could potentially be an innovative and effective approach to accelerate malaria elimination in Africa, however, this assertion is based on the epidemiological impact assessment of the intervention only. The cost-effective analysis of the project looking at the implementation of the 1,7 mRCTR approach is being looked at and it is a work in progress that will be submitted for publication.

## Conclusion

Implementation of the 1,7-mRCTR contributed convincingly to the reduction of the malaria burden in areas of moderate and high transmission in southern Tanzania and offers the first attempt at implementing surveillance as an intervention in areas with high malaria burden. Appropriately structured and defined health facility data is instrumental at allowing sub-district level microstratification and hence targeting of resources and interventions more appropriately. The results encourage a broader evaluation of the 1,7-mRCTR approach and the strategic approaches for accelerating efforts toward malaria control and elimination. Furthermore, lessons learned from the implementation of the 1,7-mRCTR approach with the community-based capacity building and local health system strengthening are shaping the Chinese aid efforts to support African countries in accelerating malaria control and elimination.

## Supplementary information

**Additional file 1:** Survey organization and Implementation.

**Additional file 2:** Analytical procedure for HFs data.

## Data Availability

All relevant data can be made available upon receipt of official requests while ensuring participant and community data privacy and confidentiality.

## Abbreviations

1,7-mRCTR: 1,7-malaria reactive community-based testing and response
cMTS: Community-based mobile test station
SSA: Sub-Saharan Africa
WHO: World Health Organization
mRDT: Malaria rapid diagnostic test
IQR: Interquartile range
aOR: Adjusted odds ratio
LLIN: Long-lasting insecticide-treated nets
CI: Confidence interval
NMCP: National malaria control programme
GEE: Generalized estimating equations
CHCWs: Community-based health care workers
HFs: Health facilities

## References

[1] WHO. World malaria report 2019. Geneva, World Health Organization; 2019. https://www.who.int/publications-detail/world-malaria-report-2019.

[2] Feachem RGA, Phillips AA, Targett GA, Snow RW (2010). Call to action: priorities for malaria elimination. Lancet.

[3] Murray CJL, Rosenfeld LC, Lim SS, Andrews KG, Foreman KJ, Haring D (2012). Global malaria mortality between 1980 and 2010: a systematic analysis. Lancet.

[4] Alonso PL, Tanner M (2013). Public health challenges and prospects for malaria control and elimination. Nat Med.

[5] Walker PG, Griffin JT, Ferguson NM, Ghani AC (2016). Estimating the most efficient allocation of interventions to achieve reductions in *Plasmodium falciparum* malaria burden and transmission in Africa: a modelling study. Lancet Glob Health..

[6] Feachem RGA, Chen I, Akbari O, Bertozzi-Villa A, Bhatt S, Binka F (2019). Malaria eradication within a generation: ambitious, achievable, and necessary. Lancet.

[7] Steketee RW, Campbell CC (2010). Impact of national malaria control scale-up programmes in Africa: magnitude and attribution of effects. Malar J..

[8] Bhattarai A, Ali AS, Kachur SP, Martensson A, Abbas AK, Khatib R (2007). Impact of artemisinin-based combination therapy and insecticide-treated nets on malaria burden in Zanzibar. PLoS Med..

[9] Bhatt S, Weiss DJ, Cameron E, Bisanzio D, Mappin B, Dalrymple U (2015). The effect of malaria control on *Plasmodium falciparum* in Africa between 2000 and 2015. Nature.

[10] Gething PW, Casey DC, Weiss DJ, Bisanzio D, Bhatt S, Cameron E (2016). Mapping *Plasmodium falciparu*m mortality in Africa between 1990 and 2015. N Engl J Med.

[11] National Malaria Control Programme (NMCP): National Malaria Strategic Plan 2014–2020. Ministry of Health and Social Welfare; Tanzania. 2014.

[12] National Malaria Control Programme (Tanzania), WHO, Ifakara Health Institute (IHI), KEMRI-Wellcome Trust (Kenya). An epidemiological profile of malaria and its control in Mainland Tanzania. Report funded by Roll Back Malaria and Department for International Development-UK; 2013.

[13] Ministry of Health Community Development, Gender, Elderly and Children (MoHCDGEC) (Tanzania Mainland), Ministry of Health (MoH) (Zanzibar), National Bureau of Statistics (NBS), Office of the Chief Government Statistician (OCGS), ICF. Tanzania malaria indicator survey 2008. Dar es Salaam, Tanzania, and Rockville, USA. 2007.

[14] Ministry of Health, Community Development, Gender, Elderly and Children (MoHCDGEC) (Tanzania Mainland), Ministry of Health (MoH) (Zanzibar), National Bureau of Statistics (NBS), Office of the Chief Government Statistician (OCGS), and ICF. Tanzania malaria indicator survey 2017. Dar es Salaam, Tanzania, Rockville, USA.

[15] WHO. Global technical strategy for malaria 2016–2030. Geneva, World Health Organization, 2016. https://www.who.int/malaria/areas/global_technical_strategy/en/.

[16] malERA Consultative Group on Monitoring E, and Surveillance. A research agenda for malaria eradication: monitoring, evaluation, and surveillance. PLoS Med. 2011;8:e1000400. 10.1371/journal.pmed.1000400 PMC3026689 21311581

[17] WHO. Test. Treat. Track: Scaling up diagnostic testing, treatment and surveillance for malaria. Geneva: World Health Organisation, 2012. https://www.who.int/malaria/publications/atoz/t3_brochure/en/.

[18] Zhou S-S, Zhang S-S, Zhang L, Rietveld AE, Ramsay AR, Zachariah R (2015). China’s 1-3-7 surveillance and response strategy for malaria elimination: is case reporting, investigation, and foci response happening according to plan?. Infect Dis Poverty..

[19] Zhang S, Zhang L, Feng J, Yin J, Feng X, Xia Z, et al. Malaria elimination in the People’s Republic of China: current progress, challenges, and prospects. In Towards Malaria Elimination-A Leap Forward. IntechOpen; 2018

[20] Cao J, Sturrock HJ, Cotter C, Zhou S, Zhou H, Liu Y (2014). Communicating and monitoring surveillance and response activities for malaria elimination: china’s “1-3-7″ strategy. PLoS Med..

[21] Feng J, Zhang L, Huang F, Yin J-H, Tu H, Xia Z-G (2018). Ready for malaria elimination: zero indigenous case reported in the People’s republic of China. Malar J..

[22] Bousema T, Griffin JT, Sauerwein RW, Smith DL, Churcher TS, Takken W (2012). Hitting hotspots: spatial targeting of malaria for control and elimination. PLoS Med..

[23] Sturrock HJ, Hsiang MS, Cohen JM, Smith DL, Greenhouse B, Bousema T (2013). Targeting asymptomatic malaria infections: active surveillance in control and elimination. PLoS Med..

[24] Sturrock HJ, Novotny JM, Kunene S, Dlamini S, Zulu Z, Cohen JM (2013). Reactive case detection for malaria elimination: real-life experience from an ongoing program in Swaziland. PLoS ONE.

[25] Landier J, Parker DM, Thu AM, Carrara VI, Lwin KM, Bonnington CA (2016). The role of early detection and treatment in malaria elimination. Malar J..

[26] Landier J, Parker DM, Thu AM, Lwin KM, Delmas G, Nosten FH (2018). Effect of generalised access to early diagnosis and treatment and targeted mass drug administration on *Plasmodium falciparum* malaria in Eastern Myanmar: an observational study of a regional elimination programme. Lancet.

[27] National Malaria Control Programme (NMCP), Tanzania. Supplementary midterm malaria strategic plan 2018–2020. Ministry of Health, Community Development, Gender, Elderly and Children; Tanzania; 2018.

[28] National Malaria Control Programme (NMCP), Tanzania (2012). Ministry of Health & Social Welfare (MoHSW) (MoHSWM).

[29] Chacky F, Runge M, Rumisha SF, Machafuko P, Chaki P, Massaga JJ (2018). Nationwide school malaria parasitaemia survey in public primary schools, the United Republic of Tanzania. Malar J..

[30] Runge M, Snow RW, Molteni F, Thawer S, Mohamed A, Mandike R (2020). Simulating the council-specific impact of anti-malaria interventions: a tool to support malaria strategic planning in Tanzania. PLoS ONE.

[31] WHO, RBM Partnership to End Malaria. High burden to high impact: a targeted malaria response. Geneva, World Health Organization; 2019. Report WHO/CDS/GMP/2018.25.

[32] Khatib RA, Chaki PP, Wang DQ, Mlacha YP, Mihayo MG, Gavana T (2018). Epidemiological characterization of malaria in rural southern Tanzania following China-Tanzania pilot joint malaria control baseline survey. Malar J..

[33] Wang D, Chaki P, Mlacha Y, Gavana T, Michael MG, Khatibu R (2019). Application of community-based and integrated strategy to reduce malaria disease burden in southern Tanzania: the study protocol of China-UK-Tanzania pilot project on malaria control. Infect Dis Poverty..

[34] Habicht JP, Victora CG, Vaughan JP (1999). Evaluation designs for adequacy, plausibility and probability of public health programme performance and impact. Int J Epidemiol.

[35] Shabani J, Lutambi AM, Mwakalinga V, Masanja H (2010). Clustering of under-five mortality in Rufiji Health and Demographic Surveillance System in rural Tanzania. Glob Health Action..

[36] National Bureau of Statistics (Tanzania), Tanzania, Office of Chief Government Statistician (Zanzibar). 2012 Population and housing census. Dar es Salaam; 2012.

[37] Ministry of Health and Social Welfare (MoHSW): National Guidelines for Diagnosis and Treatment of Malaria. Dar es Salaam, 2006.

[38] Roll Back Malaria Monitoring and Evaluation Reference Group, World Health Organization, United Nations Children’s Fund, MEASURE DHS, MEASURE Evaluation, and U.S. Centers for Disease Control and Prevention: Malaria Indicator Survey: Basic documentation for survey design and implementation. 2005, Calverton, Maryland: MEASURE Evaluation

[39] Filmer D, Pritchett LH (2001). Estimating wealth effects without expenditure data—Or tears: an application to educational enrollments in states of India. Demography.

[40] Maher B (2008). Malaria: the end of the beginning. Nature.

[41] Larsen DA, Bennett A, Silumbe K, Hamainza B, Yukich JO, Keating J (2015). Population-wide malaria testing and treatment with rapid diagnostic tests and artemether-lumefantrine in southern Zambia: a community randomized step-wedge control trial design. Am J Trop Med Hyg.

[42] Aidoo EK, Afrane YA, Machani MG, Chebore W, Lawson BW, Atieli H (2018). Reactive case detection of *Plasmodium falciparum* in western Kenya highlands: effective in identifying additional cases, yet limited effect on transmission. Malar J..

[43] Deutsch-Feldman M, Hamapumbu H, Lubinda J, Musonda M, Katowa B, Searle KM (2018). Efficiency of a Malaria Reactive Test-and-Treat Program in Southern Zambia: a prospective, observational study. Am J Trop Med Hyg.

[44] Bejon P, Williams TN, Liljander A, Noor AM, Wambua J, Ogada E (2010). Stable and unstable malaria hotspots in longitudinal cohort studies in Kenya. PLoS Med..

[45] WHO. Community-based reduction of malaria transmission. Geneva, World Health Organization, 2012. https://www.who.int/malaria/publications/atoz/9789241502719/en/.

