# Supplementary material for: Effectiveness of the innovative 1,7-malaria reactive community-based testing and response (1, 7-mRCTR) approach on malaria burden reduction in Southeastern Tanzania
Source: Malar J. 2020 Aug 14;19:292. doi: 10.1186/s12936-020-03363-w (PMC7429894; doi:10.1186/s12936-020-03363-w)
Supplement: Supplementary file 1 — Additional file 1: Survey organization and Implementation. [file 12936_2020_3363_MOESM1_ESM.doc]

### Survey organization and selection of study areas

**Introduction of the program**

This collaborative program was implemented by the Ifakara Health Institute (IHI) in Tanzania and the National Institute of Parasitic Diseases (NIPD) at China CDC. The China-United Kingdom-Tanzania pilot project on malaria control was the first project to pilot the feasibility of how Chinese models in combination with the WHO-T3 initiative could be effectively used to reduce malaria and fast track elimination efforts in Africa. The program aimed to reduce malaria disease burden by 30% in comparison with what was at the beginning of the project, through strengthening capacity for malaria control at the local level and implementation of the adopted Chinese experiences in combination with the World Health Organization T3 strategy in the proof of concept areas[1].

The design of this project started with workshops and kick-off meeting held by technical personnel from potential partners followed by field visits in Tanzania. Before study implementation, the field visits involved consultations with central and local government authorities for the identification of project sites. Based on the availability of prior data, epidemiological parameters, and logistical convenience, Rufiji district was selected. The selected study area covered four wards. Of four wards, two were assigned to the intervention arm and the other two were controls. The identification of the project sites was followed by a baseline survey to establish parameters upon which the impact of implementing the project would be evaluated. After the baseline survey, the implementation of the project started. Intervention package of the project involved the application of a modified Chinese “1-3-7” [2] model for malaria surveillance and response in combination with the WHO-T3 Initiative and the local resources. The local resources here included a platform of health system infrastructures, manpower, funds, and supplies that existed to provide the base of the intervention. Both malaria experts from China and Tanzania worked cooperatively on this community-based pilot project to provide the platform for expanding Chinese malaria experiences gained and transferred into innovative 1-7 mRCT approach development and integration in strengthening community engagement and mobilization.

**Stakeholder engagement**

The project designing and implementation was a collaborative effort by multiple individuals from local and international malaria stakeholders. At the national level, the study team was composed of members from the IHI and NIPD together with the National Malaria Control Programme (NMCP) of Tanzania and the National Institute of Medical Research (NIMR) of Tanzania. The approved protocol was shared with NMCP and NIMR for discussion and agreement on the proposed intervention. Subsequently, at the district level, the local government officers (District Medical Officer, District Malaria focal person, Vector control officer, and Council Health Management Team) were consulted and the study design was discussed in detail.

At the local community level, engagement activities were conducted before and during the screening campaigns to broaden and strengthen community awareness, to raise the general knowledge of malaria and to promote the intervention. Meetings involving; community leaders, school teachers, and children and key informants at the district, wards, villages, and sub-village levels were held.

As part of working collaboration between Chinese and Tanzania, during field implementation, at least 36 Chinese teams of malaria expertise (epidemiologist, medical entomologist, laboratory scientist, and anthropologist) were deployed at the local community to provide malaria technical and scientific assistance on study design, feasibility, and practical workflow coordination. Different from some international projects, the on-site Chinese staff for technical support were dispatched to the pilot areas and worked with local stakeholders throughout the pilot project to find out the practical issues and obstacles lying in the malaria control process and cope with them sharing Chinese experience, in a mutual learning-by-doing way. Chinese staff in the field of epidemiology, entomology, anthropology, lab sciences were selected in China nationwide to work with IHI and other local partners. Importantly, the malaria experts from China jointly working with Tanzania worked on this community-based pilot project was purposely to provide the platform for expanding Chinese malaria experiences gained and transferred into other settings like Tanzania.

The onsite Chinese staffs were twinned together with local staff from IHI, NMCP and other partners in the filed communities, jointly working on a specific aspect (e.g., surveillance & treatment, or vector control activities, etc.) for work plan designing, local staff training, field implementation, and supervision, etc. They mainly supervised and gave guidance to local training and field implementation. The working teams regularly conducted community mobilization campaigns with more than 200 local stakeholders including the local government leaders and health staff from 36 administrative villages in the 4 communities. Besides the supervision and guidance, they also ensured the steady progress of the project implementation by jointly taking solutions on the field level.

For implementing the 1,7-mRCTR, the project recruited and trained 35 community-based health care workers (CHCWs) on malaria surveillance and treatment activities. Detailed training included the fundamental skills for malaria case diagnosis, and treatment, vector control, and health education. The CHCWs were divided into four teams, which were referred to as surveillance response teams. Each surveillance response team comprising of at least one laboratory technician, a clinician, a nurse, field interviewers, and a field supervisor. The teams were deployed to the two intervention wards to conduct 1,7-mRCTR. Furthermore, a community sensitization team was formed from each respective village to raise the community’s awareness and compliance with a 1,7-mRCTR. The size of the sensitization team depended on the number of sub-villages units, including the hamlet leader and village community health volunteers.

**The role of each team member in the cMST**

Teams of CHCWs including supervisors, lab technicians, nurses, field workers were recruited from the village where this study was conducted. The CHCWs were recruited from the community to serve as a bridge between the community and the research team and other stakeholders of the project. We envisaged this approach will lead to a reduction in recruitment costs as compared with recruitment from other regions which could increase the cost to the management through additional costs such as accommodation. However, this is yet to be proved since this paper did not evaluate the cost-effectiveness of the intervention. This is planned for the next paper.

**Supervisor’s tasks:** Supervisor's responsibilities were to ensure all field workers have the necessary materials for the day, including enumeration number for the selected household (ids) to be visited by each and ensuring that every participant visiting the cMST is registered with a unique identification. Supervisors were also required to inform the pre-inform the village leaders when the village identified as hotspots. Furthermore, the supervisors had the role of ensuring that all forms were cross-checked for completeness and are accurately recorded following the standard operating procedure.

**Laboratory personnel:** This was the person responsible for receiving the samples and forms from the field, prepare the slides for reading, parasite identification, recording the results, and send the results forms to the field supervisor for further management.

**Study clinician/nurses**: The clinician's role was to review the results of participants attended at cMST and prescribe the antimalarial following the National Malaria treatment guidelines and provide necessary consultation to the participants regarding their health problems/ conditions and also to provide a referral in case of severity observation. They were also testing and ensuring that the first treatment in case of positive the antimalarial is taken at the field site.

**Field workers**: This was a frontline public health worker whose role was to administer the questionnaire and collecting all demographic details of participants attended the cMST. They were also responsible to conduct the community education on malaria prevention. Furthermore, they were also responsible for a day to day activities of ensuring that all field equipment, logistics are in place before the field to start. All of these were recruited from the village this was to ensure the trusting relationship between the research team and community members.

**References**

1. Wang D, Chaki P, Mlacha Y, Gavana T, Michael MG, Khatibu R, Feng J, Zhou ZB, Lin KM, Xia S *et al*: **Application of community-based and integrated strategy to reduce malaria disease burden in southern Tanzania: the study protocol of China-UK-Tanzania pilot project on malaria control**. *Infect Dis Poverty* 2019, **8**(1):4.

2. Zhou S-S, Zhang S-S, Zhang L, Rietveld AE, Ramsay AR, Zachariah R, Bissell K, Van den Bergh R, Xia Z-G, Zhou X-N: **China’s 1-3-7 surveillance and response strategy for malaria elimination: Is case reporting, investigation and foci response happening according to plan?** *Infect Dis Poverty* 2015, **4**(1):55.
