# Supplementary material for: Effectiveness of the innovative 1,7-malaria reactive community-based testing and response (1, 7-mRCTR) approach on malaria burden reduction in Southeastern Tanzania
Source: Malar J. 2020 Aug 14;19:292. doi: 10.1186/s12936-020-03363-w (PMC7429894; doi:10.1186/s12936-020-03363-w)
Supplement: Supplementary file 2 — Additional file 2: Analytical procedure for HFs data. [file 12936_2020_3363_MOESM2_ESM.docx]

**Statistical Analysis**

To examine whether the 1,7-mRCT affected the number of health facility cases in the treated wards, the total number of cases diagnosed at health facilities during each week for each ward were considered and computed case ratios (the number of cases at the HF per population base). Seasons were divided into the high season (weeks 18-31 of the year, roughly May to July) and low season (all other weeks) based on case count. The weekly case ratios per population base were considered as replicates of the situations within the wards. GEE models of the annual effects (2017 vs 2016 - the only full years in the project) controlled for the season were fitted. To make sure that our modeling methods did not unduly influence the results, the logarithmic case ratios (with identity link) using both independent and exchangeable correlation structures were modelled. Also, the case ratios using identity link and independent and exchangeable correlation structures was modelled. Thus, four ways of examining the outcomes were prepared. Moreover, the possible interaction of year and season, i.e. whether or not the approach tested was more or less effective depending on the season was considered.

To examine the length of time, the case ratio per population was suppressed after a village was treated, the logarithm of the case ratio using mixed models with the identity link, the exchangeable working covariance structure, and the empirical variance was modelled. Each village-week was considered to be a replicate within the village (random effect). The exposure of interest was the (integer) number of weeks post-treatment. If the time since the last treatment exceeded 13 weeks, the time was reset to ‘pre-treatment’. If a village was treated within the 13 weeks, the ‘clock’ restarted. In addition to ward and season (which was modeled harmonically), a linear term for time since the project started was included, as were interaction terms between the ward and this term as well as the season variables. Since the treatment was not allocated randomly but based on being a hotspot, we needed to weight the observations to reflect the probability that an individual village would be treated in a particular week. ^T^he method of Hernan [1] was followed to produce stabilized weights using season, ward, and linear time for the numerator model and adding the number of times a village had been previously treated, and the previous week’s incidence ratio for the denominator model. Since the stabilized weights had a large range,.005 to observations with computed weight below 0.01 and 101 to observations with computed weight over 100 was assigned.

**Reference**

1. Hernan MA, Brumback B, Robins JM. Marginal structural models to estimate the causal effect of zidovudine on the survival of HIV-positive men. Epidemiology. 2000; 11:561-570.
